# Summarizing activity limitations in children with chronic illnesses living in the community: a measurement study of scales using supplemented interRAI items

**DOI:** 10.1186/1472-6963-12-19

**Published:** 2012-01-23

**Authors:** Charles D Phillips, Ashweeta Patnaik, Darcy K Moudouni, Emily Naiser, James A Dyer, Catherine Hawes, Constance J Fournier, Thomas R Miller, Timothy R Elliott

**Affiliations:** 1 Program on Disability, Aging, and Long-Term Care Policy, Texas A&M Health Science Center, School of Rural Public Health, College Station, Texas, USA; 2 Public Policy Research Institute, Texas A&M University, College Station, Texas, USA; 3 Counseling Psychology Department, Texas A&M University, College Station, Texas, USA; 4 Child & Adolescent Health Research Laboratory, Texas A&M University, College Station, Texas, USA

## Abstract

**Background:**

To test the validity and reliability of scales intended to measure activity limitations faced by children with chronic illnesses living in the community. The scales were based on information provided by caregivers to service program personnel almost exclusively trained as social workers. The items used to measure activity limitations were interRAI items supplemented so that they were more applicable to activity limitations in children with chronic illnesses. In addition, these analyses may shed light on the possibility of gathering functional information that can span the life course as well as spanning different care settings.

**Methods:**

Analyses included testing the internal consistency, predictive, concurrent, discriminant and construct validity of two activity limitation scales. The scales were developed using assessment data gathered in the United States of America (USA) from over 2,700 assessments of children aged 4 to 20 receiving Medicaid Early and Periodic Screening, Diagnostic and Treatment (EPSDT) services, specifically Personal Care Services to assist children in overcoming activity limitations. The Medicaid program in the USA pays for health care services provided to children in low-income households. Data were collected in a single, large state in the southwestern USA in late 2008 and early 2009. A similar sample of children was assessed in 2010, and the analyses were replicated using this sample.

**Results:**

The two scales exhibited excellent internal consistency. Evidence on the concurrent, predictive, discriminant, and construct validity of the proposed scales was strong. Quite importantly, scale scores were not correlated with (confounded with) a child's developmental stage or age. The results for these scales and items were consistent across the two independent samples.

**Conclusions:**

Unpaid caregivers, usually parents, can provide assessors lacking either medical or nursing training with reliable and valid information on the activity limitations of children. One can summarize these data in scales that are both internally consistent and valid. Researchers and clinicians can use supplemented interRAI items to provide guidance for professionals and programs serving children, as well as older persons. This research emphasizes the importance of developing medical information systems that allow one to integrate information not only across care settings but also across an individual's life course.

## Background

In 2008, over 31 million children living in low-income households in the United States of America (USA) were eligible to receive services from Medicaid's Early and Periodic Screening, Diagnostic, and Treatment (EPSDT) program [1]. Medicaid in the USA uses a combination of state and federal funds to pay for health services provided to low-income families, persons with disabilities, and older persons needing long-term care. In 2009, almost 3 million children in the large, southwestern state of Texas were eligible for EPSDT services [2]. Health care for these children accounted for Medicaid expenditures of approximately US$1.8 billion [3]. The bulk of these expenditures were for the periodic examinations required for all EPSDT participants [4].

Among those in Texas receiving EPSDT services, a small but important proportion are children with chronic health problems who receive care at home. Many of these children receive assistance with activity limitations through Medicaid Personal Care Services (PCS). The average child in the Medicaid program in Texas in 2010 accumulated annual Medicaid expenditures totaling US$1,866; expenditures for children receiving PCS in their homes averaged almost 20 times that amount (US$36,314). For all children receiving home-based Medicaid services, the average annual expenditure for those home-based services was US$3,499; for those receiving PCS, the average annual bill for home-based services was US$23,469 [5].

Using the terminology of the International Classification of Function (ICF) as our frame of reference, PCS are provided by home care personnel to help individuals whose health conditions or impairments create activity limitations [6-9]. Since the 1970s, the Texas Medicaid program has, through a variety of mechanisms, provided assistance with activities of daily living (ADLs) and instrumental activities of daily living (IADLs) to low-income children whose conditions result in activity limitations. PCS are provided solely to compensate for an activity limitation deriving from a health condition. In addition, unlike some other programs, in order to receive services the adult(s) responsible for the child must be unable to provide the needed assistance [10,11].

The link between need and services is sometimes unclear, especially for children. When one observes an older person with an activity limitation, one assumes the etiology of the limitation is a health condition or impairment. An older man who could once safely bathe himself but now cannot do so is obviously in need of assistance. He may have this limitation because of an impairment (e.g., limited range of motion) created by a condition (e.g., osteoarthritis).

In contrast, a child in the EPSDT program who cannot safely bathe herself may, or may not, be suffering from a limitation due to a condition or impairment. If the child is 3 years old, an inability to bathe safely and independently is expected because of the child's developmental stage or age. Bathing a child of 3 is an activity carried out by all parents or adults responsible for a child. But, a child of 3 with Lobstein disease (brittle bone disease) may require 2-person assistance to be bathed safely or the bath may take much longer than a bath for other children of the same age. This additional care burden associated with a medical condition or impairment would make a child of any age eligible for Medicaid PCS to assist with bathing. A child of 16 with severe intellectual disability (ID) may be eligible for PCS assistance in bathing, while a child of 16 with mild ID may be perfectly capable of bathing safely and not qualify for PCS.

Other assessment tools contain the elements of the ICF model, but they are not specifically tailored to determining the need for services in the home [12]. The WeeFIM^® ^can be used to screen for developmental delay in children 6 months to 8 years old [13]. The Pediatric Evaluation of Disabilities Inventory (PEDI) can be used to evaluate the level of disability for children 6 months to 7 1/2 years old [14]. For these instruments, the determination of the presence of a limitation is based on a comparison of a child's ability with the ability of children with no known impairments and no evidence of developmental delay. These "norming" samples are, however, often of limited size and exhibit very limited diversity in race, ethnicity, and economic status [15-17].

Other instruments have different limitations. The Functional Status Scale (FSS) is designed for use with severely impaired children after hospitalization, used for a small number of activity limitations (motor function and eating), and is designed for use by highly-trained health professionals [18,19]. Other scales, like the Gross Motor Function Scale (GMFS), focus only on one aspect of function, limitations in motor function [20].

The popular Adaptive Behavior Assessment System, Second Edition (ABAS-II), in contrast assesses needs for all ages, and the validation samples are relatively large [21]. However, the coding for items in the system runs from zero (is not able to perform the task) to 3 (always or almost always performs the task). Such a scoring system does little to assist service providers in knowing how much assistance a client may need over the course of a week. Again, the focus is largely on identifying individuals with problems (e.g., those with scores two standard deviations below the norm for their group), not on determining how much assistance the child needs due to activity limitations.

This research presents the results of an effort to tailor an assessment tool to determine a child's need for home care services to address activity limitations related to their health conditions or impairments. The effort focused on the use of caregivers' reports of a child's activity limitations to an individual with no special medical, therapy, or nursing training. We based the assessment on instruments used with adults and modified items on those instruments so that they were appropriate for children. We specifically designed the instruments to recognize the reality of home care for children. In such situations, program staff and health professionals are dependent on reports from informal caregivers for much of their information about a child's limitations and strengths.

The primary goal of the effort was for EPSDT case managers in Texas to gather reliable and valid information on a child's activity limitations in order to develop a service plan for the child. Here we present information related to a secondary goal of the effort -- producing reliable and valid scales summarizing a child's health-related activity limitations.

A secondary goal of the effort, intertwined with the instrument development and scaling activities, was determining how well assessment items originally developed for use with older persons could serve as the basis for items and scales focused on children's activity limitations resulting from a health problem. This issue is of some importance for medical information systems. Most concerns about integrating medical information have related to integrating and standardizing data across care settings. Another issue, which receives less attention, is integrating medical information across a person's life course. When different information systems and measurement strategies are used for children, adults, and older persons, this reduces a clinician's ability to provide the continuity of care that can positively affect both individual and population health.

## Methods

### Participants

Since September 2007, under the leadership of the Texas Health and Human Services Commission (HHSC), case managers in the Texas Department of State Health Services (DSHS) have assessed children seeking or receiving EPSDT services to determine their need for PCS. In September 2008, case managers began using new assessment tools. Two multi-dimensional assessment instruments were developed and tested for reliability and face validity prior to their implementation. The Personal Care Assessment Form 0-3 (PCAF 0-3) was used to assess the PCS need of children under 4 years old. The Personal Care Assessment Form 4-20 (PCAF 4-20) was used to assess all other children seeking or using PCS as a part of EPSDT services.

For the first 6 months of the PCAF's use, case managers submitted all completed PCAFs to the research team. Nine of the 11 state health regions provided PCAF data from September 2008 through February 2009. Implementation was delayed in 2 regions because of the demands placed on DSHS staff by hurricane damage. These regions supplied data from December 2008 through March 2009. These paper forms were reviewed and entered into an electronic database.

The research team received 3,068 assessments. A few assessments (8) could not be used due to missing data, and 99 assessments involved no authorization for PCS services. The PCAF 0-3 data included 201 children. In Texas, 5,493 children with chronic illnesses were participating in the PCS program in April, 2009 when all data collection ended. Our sample represents just over one-half of the population of children receiving PCS. Ninety-three percent of those assessed were over 3 years of age. This research is restricted to the 2,760 children from 4 to 20 years old.

The assessment instruments were also used by Medicaid managed care organizations (MCOs), but these organizations did not provide assessment data to the State or the research team. Thus, the study population was restricted to those children in the Medicaid Fee-For-Service market or children in the Medicaid Primary Care Case Management Program. This restriction does no harm to the study. This research investigates functional measurement; the goals do not include providing population estimates for the entire Medicaid population.

PCAFs were completed in a home visit in which the primary caregiver and the child were present. The assessor was a case manager employed by the Texas Department of State Health Services. A few case managers were nurses, but licensed social workers with a master's degree in social work (MSW) comprised the vast majority of case managers. The assessment was completed by using the instrument to query the caregiver, informal observation of the child, and questioning the child (if the child was capable of responding).

An almost identical data collection was completed in 2010, after minor changes unrelated to the measures of activity limitations were made in the instrument. This effort lasted four months, and PCAF data were collected in early 2010 on a sample of 2,642 children in the EPSDT program across Texas. All research activities in the project were approved by the Institutional Review Board of Texas A&M University.

### Measurement

The PCAF was purpose-built for determining children's needs for PCS. It was largely based on items, or variants of items, included in the Minimum Data Set for Nursing Home Resident Assessment (now the interRAI-LTCF^©^) [22] or the Minimum Data Set for Home Care^©^(now the interRAI-HC^©^) [23]. Both instruments were developed for use with frail older persons and have proven reliability and validity when used with older populations [24-26]. For an older population, the interRAI ADLs exhibit excellent reliability and scale very well [27]. The research team modified items, response sets, and examples on these assessment tools for adults so that they were appropriate for use with children.

The PCAF relies heavily on input from the primary caregiver, usually a family member. In order to receive Medicaid Personal Care Services, a child in the EPSDT program must have a primary caregiver who provides assistance with activity limitations. PCS services can be provided only when the caregivers, or caregivers, have some barrier to providing needed assistance.

The PCAF included items on the assistance provided over the last 7 days in bed mobility, positioning while upright, eating, transfer, mobility inside the dwelling, mobility outside, toilet use, dressing, personal hygiene, and bathing. Initial testing of these items with children receiving EPSDT services resulted in weighted kappas for the individual ADL items that ranged from 0.55 to 0.74, with an average of 0.64 [28]. The kappas indicated that all 10 items exhibited moderate (4 items) or substantial (6 items) reliability [29]. The reliability test involved 264 dual assessments.

While some instruments use a 3-day look-back window, for children the research team decided that a 7-day period was more appropriate. Often parents and other informal caregivers are at work or school during weekdays, and the child is usually at school during weekdays. Weekends are the times that informal caregivers get a more detailed picture of the child's needs. In support of this assumption, our analyses of the 2009 data indicate that formal care time varied little across the weekdays, but formal care hours changed significantly on Saturday and Sunday.

With the exception of bathing, the ADL items used the same response set:

• total independence;

• set-up help only;

• monitoring/supervision/cueing/redirection with rare hands-on assistance;

• limited assistance, regular hands-on assistance with rare weight-bearing assistance;

• extensive assistance, some involvement by the participant but regular weight-bearing help from a caregiver; or

• total dependence, the activity was performed by the caregiver with no or minimal assistance from the child.

The codes for bathing also ranged from 0 to 5, but the response set differed slightly from the other ADLs.

Like all interRAI activity limitation items, the caregiver indicated the assistance provided with each ADL. For children, the assessor also determined, through discussions with the caregiver and observation, whether a child's activity limitation was affected by her or his health conditions. Those children whose performance in an activity was not affected by a condition were classified as independent in our analyses. Those whose ability to perform an activity was affected by a health condition or impairment kept the assistance score originally assigned for that activity.

Thus, a child of 4 might be coded as totally dependent in dressing. However, if this need was not driven by a health condition, but simply by developmental stage or age, then in our analyses, the child's was scored as being independent. Children with an activity limitation that derived from an underlying impairment or condition received the score that reflected how much care they received with that activity (e.g., limited assistance, total dependence). In each ADL, the child's score depends on the impact of their health conditions or impairments on their activity limitations. The score is not a function of the child's developmental stage or age.

For illustrative purposes, this research focuses on 2 activity limitation scales. An additive scale for all 10 activity scores (0-50) is the first. The second is an additive scale (0-10) reflecting the number of activities in which the child regularly received at least hands-on assistance (i.e., limited assistance, extensive assistance, or total dependence). The research team developed both scales; case managers had no ADL summary measures available to them when they determined the child's service needs.

The chosen measurement strategy demands that caregivers made judgments about whether a child's activity limitations derive from a condition or impairment. We investigate the validity and reliability of activity limitation scales derived from the items that demand this judgment. In doing so, we provide one test of the reliability and validity of caregivers' judgments about the etiology of children's activity limitations.

Other PCAF items were used in exploring the usefulness of this measurement strategy and the scales summarizing a child's limitations. These items included information on the child's age, cognitive status, and performance in IADLs, and the number of hours of PCS authorized following assessment. A complete copy of the PCAF 4-20 is available at http://pcaf.tamu.edu.

### Analyses

The analyses focused on the distributional and psychometric properties of our two scales. We tested the internal consistency of the scales for different portions of the sample. The predictive, discriminant, concurrent, and construct validity of the scales were tested using other items in the PCAF [30-32]. The tests were initially performed using the 2008-2009 database. The 2008-2009 results were then validated using assessments in the 2010 database for children not included in the earlier wave of assessments.

## Results

Table 1 presents descriptive statistics for our study participants. The majority was male, and sample members were relatively evenly spread across the age span covered by the sample. Almost two-thirds of these children faced multiple challenges, with 64 percent presenting with both medical and mental health conditions. Almost one-half of them had an intellectual disability of some type; one-quarter had attention deficit hyperactivity disorder (ADHD); over one-quarter had a seizure disorder. Four out of 5 study participants had significant difficulty following multi-step instructions.

**Table 1:** Descriptive data for Children with Chronic Illnesses Living in Low-Income Households (n = 2,755)

Variables	Categories	Prevalence (%)
Gender	Male	57.7

Age group	4-8 years old	29.5

	9-12 years old	24.4

	13-16 years old	21.1

	17-20 year old	25.0

Child's current qualifying condition(s)	Medical only	22.0

	Psychiatric/developmental/behavioral only	14.0

	Both types of conditions	64.0

Most Commonly Reported Diagnoses Or Conditions (Prevalence 10% or greater)	Epilepsy/chronic seizure disorder	28.8

	Asthma/respiratory disorder	24.8

	Cerebral palsy	24.4

	Paraplegia/tetraplegia/quadriplegia	11.2

	Intellectual disability	48.4

	ADHD	25.7

	Autistic disorder	16.7

	Mood disorder	14.1

	Anxiety disorder	13.6

	Disruptive behavior disorder	10.6

	Restricted range of motion	35.3

	Pain	17.1

	Bedbound	16.2

	Contractures	14.9

	Falls related to condition	13.5

Procedural memory	Unable to follow most or all multi-step instructions	82.3

Receptive Communication	Sometimes, rarely, or never understands	54.8

Bladder control	Frequent, usually, or always incontinent	47.5

Control of bowels	Frequent, usually, or always incontinent	43.5

Urgent medical care in 30 days prior to assessment	Physician visit	11.1

	Emergency department	8.0

	Hospital admission	5.3

The participants' activity limitations appear in Table 2. Sample members were least likely to need hands-on assistance with bed mobility and most likely to need it with bathing. The degree to which activities were affected by the child's conditions and the degree to which they required hands-on assistance break the activities into 3 groups. *Very Complex *activities (toilet use, personal hygiene, dressing, bathing), most likely to require personal care, involved multiple steps and needed fine motor control. *Less Complex *activities, least likely to require assistance, involved a limited number of ordered steps or the use of large muscle groups (bed mobility, locomotion inside, and positioning). Between these two groups are activities that call for gross motor control but do involve a number of ordered tasks; these are simply classified as C*omplex *(locomotion outside, transfer) [33].

**Table 2:** Activity Limitations among Children with Chronic Illnesses Living in Low-Income Households (n = 2,755)

Activity	% Whose Performance Was Affected by Their Condition(s)	% Who Needed Hands-on Assistance
Bed mobility	30.1	27.8

Positioning	32.9	30.3

Locomotion inside	38.4	33.6

Transfer	40.8	38.2

Locomotion outside	46.6	40.7

Eating	59.2	42.4

Toilet use	83.0	72.2

Personal hygiene	91.9	79.0

Dressing	92.1	79.6

Bathing	94.6	82.8

Table 3 presents information on the distribution of the scales and some of their psychometric properties. The two scales were relatively well distributed with little skewness, but their distributions were a bit flattened at their high point (negative kurtosis). The coefficient of variation for both scales was roughly 0.60. The average score on the Additive Scale was 25, while the Hands-on Scale indicated that the average sample participant needed hands-on assistance with 5 of 10 activities. The inter-quartile range for the Additive Scale was from 13-40. On the Hands-on Scale, the inter-quartile index indicated that one-quarter of the sample needed hands-on assistance in fewer than 3 activities, while another quarter received hands-on assistance in 9 or 10 activities. Both scales had excellent internal consistency (α ≥ 0.90). This strong internal consistency carried over to subgroups in the sample, specifically for both genders and within each of our 4 age groups. The analyses of internal consistency also focused on the degree to which the scale held together for three different sub-populations - those children with medical problems alone, those with only psychiatric, developmental or behavioral problems, and finally, the majority, who exhibited both classes of problems.

**Table 3:** ADL or Activity Limitation Scales based on PCAF Activity Limitation Measures

Scale Properties	Development Sample, 2009 (n = 2760)	Development Sample, 2009 (n = 2760)	Validation Sample, 2010 (n = 1765)	Validation Sample, 2010 (n = 1765)
	**HC-PEDS** **ADL Limitations Scale**	**HC-PEDS** **Hands-on Needs Scale**	**HC-PEDS** **ADL Limitations Scale**	**HC-PEDS** **Hands-on Needs Scale**

**Distribution**				

Mean	24.9	5.3	23.0	4.9

St. Deviation	15.3	3.3	14.3	3.1

Skewness	0.4	0.1	0.6	0.2

Kurtosis	-1.1	-1.1	-0.8	-0.9

Median	21	5	19	4

Inter-quartile range	13-40	3-9	12-33	3-8

Range	0-50	0-10	0-50	0-10

**Internal Consistency (unstandardized Cronbach's alpha)**				

Full sample	0.93	0.90	0.93	0.89

Ages 4-8	0.92	0.88	0.91	0.86

Ages 9-12	0.93	0.90	0.93	0.89

Ages 13-16	0.94	0.91	0.93	0.90

Ages 17-20	0.93	0.90	0.94	0.88

Male	0.93	0.90	0.93	0.89

Female	0.93	0.90	0.92	0.89

Medical conditions only	0.93	0.89	0.92	0.88

Psychiatric, development or behavioral conditions only	0.79	0.76	0.77	0.73

Both medical and psychiatric, development or behavioral conditions.	0.93	0.90	0.93	0.89

**Discriminant Validity (Pearson's r)**				

Age	-0.016 (p = 0.39)	-0.012 (p = 0.50)	-0.0174 (p = 0.46)	-0.025 (p = 0.28)

**Concurrent Validity (Pearson's r)**				

Additive IADL scale (2009 sample α = 0.88; 2010 sample α = 0.87)	0.329	0.304	0.320	0.292

2-Person Assistance w/an ADL	0.464	0.444	0.538	0.504

Cognitive Scale (2009 sample α = 0.82; 2010 sample α = 0.82)	0.355	0.309	0.368	0.315

**Predictive Validity (Pearson's r)**				

PCS hours authorized	0.455	0.435	0.436	0.424

**Construct Validity (R^2^)**				

OLS Model	0.65	0.59	0.71	0.64

For the purposes of establishing an important aspect of discriminant validity, we analyzed the relationship between scale scores and age. For those interested in screening for developmental delay, the correlation of a screening tool score and age should be strong, as it is with the WeeFIM^® ^[13]. For a measure of activity limitations to determine service needs, the measure should not correlate with age. We are seeking measures of a child's service needs that are not intertwined with a child's age or level of development.

Though other aspects of discriminant validity might be considered, the lack of significant correlations between the 2 scales and the age of the children in the sample provides crucial evidence of an important aspect of the scales' discriminant validity. The lack of a correlation between age and the scales implies that the supplemented interRAI items are measuring activity limitations that derive from health conditions or impairments, not a child's age or developmental stage.

For concurrent validity, one hopes to correlate the scales under scrutiny with established scales measuring the same construct. However, in the project team's field data collection, which was dependent on the staff and resources of the DSHS, the use of multiple items to measure the same construct (activity limitations) was not feasible. Instead, the measures we must use to test for one aspect of concurrent validity are other PCAF items that should be related to a child's functional status and any ADL scale. In this instance, we correlated the 2 ADL scales with an IADL scale (a scale summarizing a child's needs for assistance in 7 instrumental activities of daily living) and a binary item indicating whether the child needed 2-person assistance with any activity. As should be the case, both scales exhibit significant positive correlations with these alternative measures of the severity of activity limitations. The scales also exhibited a strong positive correlation with our 5-item, additive cognitive function scale composed of items related to short-term memory, long-term memory, decision-making, receptive communication, and expressive communication.

The predictive validity of our activity limitation scales was tested by correlating the scale values with the number of hours of weekly PCS authorized by the DSHS case manager following the assessment. These correlation coefficients were well in the range (0.40-0.50) of correlations observed when evaluating the correlation of activity limitations with service provision for other populations [34,35].

Construct validity demands that a scale fit into a conceptual framework or theoretical schema [32]. While we lack some explicit deductive theory into which we can place scales measuring a child's activity limitations, a conceptual framework is available. Within the ICF framework, activity limitations should, in part, be a function of diseases and disorders and their effects on bodily structures or function [7]. To put it in other terms, a scale measuring activity limitations should serve as a statement of the totality of the effects of health conditions or impairments on a child's activity limitations [36]. With chronic illnesses, one frequently observes specific conditions that create an array of impairments and activity limitations. These conditions create a cascade of problems.

For example, paralysis can be the result of a variety of diagnoses. No matter the condition, paralysis can reduce respiratory function, impair cardiovascular function, or facilitate skin breakdown. These impairments can result, respectively, in recurrent lower respiratory infections, dyspnea, or soft tissue infections. In instances where a child has multiple conditions or impairments, one's ability to attribute limitations to any specific health challenge is difficult. So a scale reflecting activity limitations serves as a convenient summary of the totality of the effects of a person's heath conditions and impairments on their ability to perform basic life skills.

To test the construct validity of the 2 scales, we used the scales as dependent variables in ordinary least squares (OLS) regressions, using health conditions and impairments as independent variables. These problems included the child's:

• cognitive function -- a scale based on items related to short-term memory, long-term memory, interpretive communication, executive function, and procedural memory.

• problems with urinary, bowel or night-time continence -- an indicator of any continence problem

• medical co-morbidities and impairments -- a binary indicator for the presence of a variety of condition or impairments that might affect ADL function.

• type of condition that created the need for assistance -- behavioral, psychiatric, or developmental conditions alone; medical conditions only; or both medical and psychological conditions.

All these data came from the PCAF assessment (see http://pcaf.tamu.edu).

The R^2 ^for those models varied from 0.71 to 0.59. These scales summarizing limitations are driven, as one would hope, largely by the child's conditions and impairments.

As columns three and four indicate, the results obtained in our analyses of the 2008-2009 data were duplicated when the same analyses were performed on a similar database of children assessed for the PCS program for the first time in early 2010. The results in the first two columns of Table 3 are not idiosyncratic.

## Discussion

One goal of this effort was to produce measures of the health-related activity limitations for children facing chronic illness. We constructed these indicators and scales so that they were consistent with the ICF framework and consistent with interRAI instruments coming into greater use in the United States and internationally. The items also met the requirement that any activity limitation results from a medical condition or impairment.

The scales developed from the individual items related to ADL performance had very good internal consistency. This was true for the population as a whole and for a number of sub-groups in the population. The scales also demonstrated good to excellent concurrent, predictive, and construct validity. The results demonstrate that low-income caregivers reporting to state program staff with no medical or nursing training can provide valid and reliable data on children's activity limitations and strengths. All these results were consistent across both the developmental and the validation samples, indicating that the scales may have some measure of external validity.

In addition, the two scales exhibited an important aspect of discriminant validity. A child's score on these scales was independent of the child's age. A strong correlation with age is a laudatory quality in an instrument designed to screen for developmental delay [13]. However, when seeking to measure activity limitations that derive from health conditions or impairments, such a correlation is unfortunate. It negates one's ability to make the important claim that the measured limitations result from the child's health, not the child's age or developmental stage. Finally, this approach to measuring activity limitations required neither reference to potentially questionable and culturally-biased population norms nor an assessment by highly- trained, highly-paid medical or nursing professionals, which can be in short supply in some settings.

The findings in this research also imply that while the size and proportions of the body change, bathing for a 10 year old or a 70 year old involves the same mechanics. In both instances, we can measure the assistance provided on the same metric. Measures with the same logic and response sets can be valid and reliable when used to assess activity limitations in a child or an older person. This indicates that clinicians and researchers may be able to measure functionality with almost identical assessment tools and discuss activity limitations in a common language throughout an individual's life course. The results imply that not only can we have a "common language" for discussing activity limitations across care setting, but we may also be able to use that language across a person's lifespan.

This research, of course, has limitations. These data come from a single state. They are restricted to those children receiving Medicaid services outside managed care organizations. All participants came from low-income households, and many were members of ethnic or racial minorities. If the underlying dynamics of caregiving and functional loss differ in other populations or settings, then these results may have limited external validity. Also, the tests for concurrent validity in this research were limited by the available data. Additional research to fill these gaps would seem to be the next logical steps in this area of research.

## Conclusions

These results clearly reinforce the idea that home care is a partnership between providers and families. Our main source of information on a child's activity limitations was an unpaid caregiver, usually a family member. This person usually has the most detailed information about a child's limitations [36]. Even for the most impaired children living in the community, family and informal caregivers provide the bulk of the care [37]. This research indicates that information provided by family caregivers to non-medical professionals forms a firm foundation for the construction of a personalized treatment plan aimed at ameliorating the effects of a child's health problems on their physical activity limitations [36,38]. Such comprehensive sources of health status collected regularly can also have salutary effects of a population's health. For example, considerable evidence suggests the use of such a comprehensive regularized assessment process improved the quality of care provided to nursing home residents in the USA [22,27].

Our efforts also have implications for those committed to unified health information systems [39]. Much of the discussion of information integration has focused on integrating information across care settings. This research focused on a different issue - the possibility of integrating health status information across an individual's life course. Our results indicate that the measures of activity limitations that work well with the elderly can also, with appropriate supplementation and minor revision, provide reliable and valid information on the needs of children. Within a specific cultural matrix (e.g., industrialized western societies), activities of daily living are essentially the same for children and adults. Such a system is important for all persons seeking health care, but it is of crucial importance for the provision of high quality care to individuals with lifelong chronic illnesses.

## Competing interests

The authors declare that they have no competing interests.

## Authors' contributions

CDP, EN, JAD, CH, CJF, and TRE developed the instrumentation and project plan. JAD and EN supervised the data collection. AP cleaned the data. All authors were involved in planning the paper and analyses of the results. AP, DKM, and TRM performed the analyses. CDP wrote the first draft. All authors commented on and approved the final draft of the paper.

## Pre-publication history

The pre-publication history for this paper can be accessed here:

http://www.biomedcentral.com/1472-6963/12/19/prepub
